# The Cost-Effectiveness of Intermittent Preventive Treatment for Malaria in Infants in Sub-Saharan Africa

**DOI:** 10.1371/journal.pone.0010313

**Published:** 2010-06-15

**Authors:** Lesong Conteh, Elisa Sicuri, Fatuma Manzi, Guy Hutton, Benson Obonyo, Fabrizio Tediosi, Prosper Biao, Paul Masika, Fred Matovu, Peter Otieno, Roly D. Gosling, Mary Hamel, Frank O. Odhiambo, Martin P. Grobusch, Peter G. Kremsner, Daniel Chandramohan, John J. Aponte, Andrea Egan, David Schellenberg, Eusebio Macete, Laurence Slutsker, Robert D. Newman, Pedro Alonso, Clara Menéndez, Marcel Tanner

**Affiliations:** 1 Swiss Tropical and Public Health Institute, Basel, Switzerland; 2 Barcelona Centre for International Health Research (CRESIB), Hospital Clínic/Institut d'Investigacions Biomèdiques August Pi i Sunyer, Universitat de Barcelona, Barcelona, Spain; 3 CIBER Epidemiología y Salud Pública (CIBERESP), Barcelona, Spain; 4 Ifakara Health Institute (IHI), Dar es Salaam, Tanzania; 5 Kenya Medical Research Institute, Centre for Global Health Research, Kisumu, Kenya; 6 University of Boconni, Milan, Italy; 7 Consultant, Cotonou, Benin; 8 National Institute for Medical Research, Tanga, Tanzania; 9 Faculty of Economics and Management, Makerere University, Kampala, Uganda; 10 London School of Hygiene and Tropical Medicine, London, United Kingdom; 11 Malaria Branch, Division of Parasitic Diseases, Centers for Disease Control and Prevention, Atlanta, Georgia, United States of America; 12 Medical Research Unit, Albert Schweitzer Hospital, Lambaréné, Gabon; 13 Division of Infectious Diseases, Faculty of Health Sciences, School of Medicine, University of the Witwatersrand, Johannesburg, South Africa; 14 Institute of Tropical Medicine, University of Tübingen, Tübingen, Germany; 15 Manhiça Health Research Center, Manhiça, Mozambique; The George Washington University Medical Center, United States of America

## Abstract

**Background:**

Intermittent preventive treatment in infants (IPTi) has been shown to decrease clinical malaria by approximately 30% in the first year of life and is a promising malaria control strategy for Sub-Saharan Africa which can be delivered alongside the Expanded Programme on Immunisation (EPI). To date, there have been limited data on the cost-effectiveness of this strategy using sulfadoxine pyrimethamine (SP) and no published data on cost-effectiveness using other antimalarials.

**Methods:**

We analysed data from 5 countries in sub-Saharan Africa using a total of 5 different IPTi drug regimens; SP, mefloquine (MQ), 3 days of chlorproguanil-dapsone (CD), SP plus 3 days of artesunate (SP-AS3) and 3 days of amodiaquine-artesunate (AQ3-AS3).The cost per malaria episode averted and cost per Disability-Adjusted Life-Year (DALY) averted were modeled using both trial specific protective efficacy (PE) for all IPTi drugs and a pooled PE for IPTi with SP, malaria incidence, an estimated malaria case fatality rate of 1.57%, IPTi delivery costs and country specific provider and household malaria treatment costs.

**Findings:**

In sites where IPTi had a significant effect on reducing malaria, the cost per episode averted for IPTi-SP was very low, USD 1.36–4.03 based on trial specific data and USD 0.68–2.27 based on the pooled analysis. For IPTi using alternative antimalarials, the lowest cost per case averted was for AQ3-AS3 in western Kenya (USD 4.62) and the highest was for MQ in Korowge, Tanzania (USD 18.56). Where efficacious, based only on intervention costs, IPTi was shown to be cost effective in all the sites and *highly* cost-effective in all but one of the sites, ranging from USD 2.90 (Ifakara, Tanzania with SP) to USD 39.63 (Korogwe, Tanzania with MQ) per DALY averted. In addition, IPTi reduced health system costs and showed significant savings to households from malaria cases averted. A threshold analysis showed that there is room for the IPTi-efficacy to fall and still remain highly cost effective in all sites where IPTi had a statistically significant effect on clinical malaria.

**Conclusions:**

IPTi delivered alongside the EPI is a highly cost effective intervention against clinical malaria with a range of drugs in a range of malaria transmission settings. Where IPTi did not have a statistically significant impact on malaria, generally in low transmission sites, it was not cost effective.

## Introduction

Malaria continues to devastate lives: 247 million malaria cases were reported among 3.3 billion people at risk in 2006 mostly in sub-Saharan Africa, and mostly in children under five [1]. One promising prevention strategy is intermittent preventive treatment of malaria in infancy (IPTi), which involves delivering treatment doses of an antimalarial drug at specified times during routine Expanded Programme on Immunization (EPI) visits, regardless of *Plasmodium* infection status [2], [3].

A pooled analysis of data from 6 completed trials of IPTi with sulfadoxine-pyrimethamine (SP) demonstrated 30% (20%; 39%) protective efficacy (PE) against clinical malaria, 38% (13%; 56%) PE against hospitalisations with malaria parasites, 23% (10%; 34%) PE against all-cause hospital admissions and 21% (8%; 33%) PE against anaemia in the first year of life[4]. Two further IPTi studies used drugs other than SP. In northern Tanzania, IPTi with mefloquine (MQ) was shown to reduce episodes of malaria in infants in a moderate transmission setting (PE 38%)[5], but had no protective effect against other outcomes including anaemia and hospital admission. Neither IPTi with SP nor 3 days of chlorproguanil-dapsone (CD3) were efficacious in this site. In western Kenya the PE of SP plus 3 days of artesunate (SP-AS3) and 3 days of amodiaquine-artesunate (AQ3-AS3) were 22% and 25% respectively against all episodes of malaria during the first year of life [6]. Three days of CD had no significant protective effect in this site either.

In addition to the efficacy of IPTi, the costs associated with introducing and successfully delivering the intervention as part of an integrated health system have been studied in detail [7], as have the policy implications of introducing and sustaining the delivery of IPTi [2], [8], [9] and the perceptions of the communities who have received IPTi[10], [11]. To date, there has only been one evaluation of the cost-effectiveness of delivering IPTi, in Ifakara, Tanzania and Manhica, Mozambique, which showed IPTi to be highly cost-effective in both settings, at under USD12 per Disability-Adjusted Life-Year (DALY) averted [12].

In this paper, we report on the cost-effectiveness of IPTi across all IPTi clinical trial study sites in sub-Saharan Africa, spanning nine sites in five countries using five different drug regimens.

## Methods

### Ethics Statement

The study was approved by the ethical committees of the various research institutes associated with this work. After obtaining written, informed consent, data on malaria treatment costs were collected from the families of children involved in the study. In an attempt to assess the resource use and associated costs of treating children with malaria from the health facility perspective, written or verbal consent was sought from the health care professionals before they were interviewed, or observed, depending on site specific requirements. Verbal consent was considered sufficient for health care workers in certain sites as the socio economic analysis came at the end of the wider efficacy study, therefore facility staff had a history of working with those in the study and were already sensitized to the aims and objectives of the cost effectiveness sub-study.

### Study Settings

Table S1 offers an overview of the characteristics of the IPTi studies included in the economic analysis. Costing data was collected in association with all the trials. More detailed explanations of the study characteristics can be found elsewhere: Ifakara, southern Tanzania [13], Manhica, Mozambique [14], Lambaréné, Gabon [15], Korogwe in Tanga region and Same in Kilimanjaro region, northern Tanzania[5] western Kenya [6] and the 3 Ghanaian sites Tamale [16], Kumasi [17] and Navrongo [18]. For the 3 Ghanaian IPTi studies the costs were associated with treating infant inpatient and outpatient malaria collected alongside a seasonal IPT for children trial in Ghana [19]. The same economist and costing methodology were used in Ghana as in this analysis.

### Effectiveness Data

Local conditions and logistical considerations led to slight differences in the methods used to detect clinical outcomes of interest across the studies, however, in general, similar and therefore comparable effectiveness outcomes were collected across the sites. The cost-effectiveness ratios presented include cost per malaria episode averted and cost per Disability-Adjusted Life-Year (DALY) averted [20], [21], [22]. DALYs averted are calculated by combining burden of disease averted from less malaria morbidity (as a function of malaria incidence, length of disease, and impact on quality of life) and less malaria mortality (as a function of malaria incidence, case fatality rate (CFR) and country specific average life expectancy at age one year [23]). In this analysis, to estimate the potential DALYs averted an assumed base CFR from malaria in infants of 1.57% was taken from a recent epidemiological model based on field data [24], [25] used in a previous cost-effectiveness analysis (CEA) of IPTi [12]. DALYs were calculated excluding age weighting, using a 3% discount rate and the disability weights given in the Global Burden of Disease study [26]. Table S2 summarises the effectiveness and health seeking inputs.

### Costings

The Cost Effectiveness Working Group (CEWG) was part of the IPTi Consortium and responsible for coordinating the collection and/or analysis of primary cost data in Kisumu, Korogwe, Same, Lambaréné, Manhica, Ifakara and Mtwara. All costs are presented in 2007 USD.

#### Costs of intervention

The costs of the intervention were based on a detailed costing of IPTi delivery in Mtwara, southern Tanzania where 13,976 infants were given IPTi over the course of 2 years as part of a phased implementation study delivering IPTi within routine health services across five districts [8]. A breakdown of the cost per dose of delivering SP in Mtwara is given elsewhere [7]. Costs included national and district costs associated with: policy change; community sensitization; behaviour change and communication; drug purchase and distribution; training; administration of IPTi in health facilities and management. The integration of IPTi delivery into the existing health system structures and functions varied by site. For example in Gabon the dispensing of IPTi was undertaken in the research centre separate from the main health facilities, whereas in western Kenya, IPTi was distributed with EPI vaccinations on a daily basis in dispensaries, health centers, and the outpatient department of a mission hospital. For the purposes of this paper, the costs of delivering IPTi in Mtwara were adapted to the various different study sites to represent the cost of delivering IPTi in operational circumstances and not one generated in more artificial trial conditions. In the cost analysis of the Mtwara study, international drug prices were used [7], whereas in this analysis the SP and CD drug prices from the local or national government central medical stores were available and therefore used. The costs of SP-AS, AQ-AS and MQ were identified on the International Drug Price Indicators List [27] or a joint UN Agencies price list [28]. Both tradable and non tradable components of the cost of delivering IPTi were adjusted to USD 2007. USD inflation rates were used for the tradable component (drug costs). The non tradable components of the unit costs (for example resources associated with community sensitisation and training) were adjusted based on international dollar differences using purchasing power parity (PPP) adjustment rates, to work out the international dollar equivalent of buying the same amount of goods and services outside Tanzania [29].

#### Provider costs associated with malaria treatment

The economic costs to providers of treating malaria were based on detailed retrospective cost data from health facilities across the study sites between 2006 and 2007. A standardised costing template was used in all the sites to record resource use associated with personnel, materials and supplies, equipment, transport, utilities and buildings. Costings were undertaken at primary, secondary and tertiary level health facilities. Costs were identified using information found in patient folders, facility stock records, activity data collected as part of health information measurements systems, discussions with health facility personnel (both medical and administrative) and components of the IPTi study budgets. A standard ingredients approach was used which involved costing the quantity used and the value of each unit of input needed to provide an inpatient or outpatient visit to treat malaria [30], [31].

#### Household costs associated with malaria treatment

Costs incurred at the household level were collected through structured exit interviews. These were administered to caretakers of children as they left health facilities after an inpatient or outpatient visit which had been categorised as malaria. A minimum of 150 inpatient and 150 outpatient interviews were conducted in each CEWG site. All participants gave informed consent. We gathered data on both indirect and direct costs incurred by patients' families. Direct costs included out-of-pocket expenses such as hospital fees, as well as expenditure on items such as food and transport. Drugs prescribed to treat malaria were identified using a mixture of data from the exit interviews and records of study patients who had visited inpatient or outpatient care. These drugs were then costed using cost schedules identified at the district hospital pharmacy and the district health directorate store. Care was taken to correctly categorise which drug costs were borne by the provider and which by the household. Indirect costs included salary lost as a result of time caring for a sick child at home, traveling to hospital, and the time spent at the facility while the infant in their care was receiving treatment. Table S3 summarises cost inputs.

### Cost-effectiveness model

The approach presented in this paper is based on the model used by Hutton et al. in their CEA of IPTi in Ifakara, Tanzania and Manhiça, Mozambique [12]. Three factors account for the slightly different incremental cost-effectiveness ratios (ICERs) in this analysis compared to those in Hutton et al. In this analysis we used slightly different (i) hospitalisation rates (ii) treatment seeking behaviour estimates and (iii) provider and household costs due to more recent cost estimates for Ifakara and inflation for Manhica. A reference target population of 1000 immunized infants were the base for calculating the aggregate effect of the IPTi intervention. This was then divided by the estimated aggregate cost of providing the intervention to give the ICERs, reflecting the IPTi intervention compared to current practice without IPTi.

Two types of data were used to estimate cost per case averted. The first was based on cost and efficacy data taken directly from each of the trials to reflect the intervention period. Table S1 outlines the variation in timing and number of IPTi doses across the trials. We used the incidence of malaria as measured in the placebo arm of each trial. In trials using SP, a second set of ICERs used the costs from each trial that were associated with delivering all IPTi doses up to twelve months of age, and the efficacy results of the pooled analysis of these 6 trials [4]. The combined estimate using random effects meta-analysis of 30.3% reflected the PE of IPTi-SP against all clinical episodes of malaria up to one year based on data from Manhica, Lambaréné, Ifakara, Navrongo, Kumasi and Tamale. The site-specific incidences of malaria up to 12 months of age presented as part of the pooled analysis were also used [4]. In both site-specific and pooled analyses, the cost savings to the public health system were based on site-specific (western Kenya, Same, Korogwe) or country-specific (Manhica, Lambaréné, Ifakara, Navrongo, Kumasi and Tamale.) estimates of the proportion of children under the age of five years, with suspected episode of malaria, who access government facilities. Severe episodes of malaria were based on the incidence of hospital admission with malaria parasites presented in the pooled analysis for SP sites and site specific publications for non SP IPTi [32]. It was not possible to find an exact definition of severe malaria that was common among the trials and therefore a proxy of hospital admission with malaria parasites was used. We recognise that this is unlikely to strictly equal severe malaria because causality is not evaluated. However this was used in the absence of better data. This assumption influences the cost savings to providers and households and it is not an input used to determine the ICERs based on intervention costs. Table S2 presents these inputs.

Results are presented from four perspectives: (1): gross intervention costs: total IPTi intervention costs, (2) net intervention costs: health system costs savings due to less malaria treatment seeking at government health facilities are subtracted from gross intervention costs (3) total societal direct cost savings: direct patient cost savings due to less malaria treatment and; (4) total societal indirect cost savings: household cost savings associated with a reduction in loss of productivity when caring for a sick child due to less malaria treatment.

#### Uncertainty

ICERs were calculated as probability distributions rather than as point estimates. Ranges used for the input variables were calculated in different ways and triangular distributions were assigned [33]. When available, the original trial data confidence intervals were used, as for the PE. Ranges for the CFR (1% to 3%), malaria incidence and IPTi intervention costs are based on the range variability as represented in Hutton et al. [12]. In sites where provider costs were not available, range variability from western Kenya was used. In sites where ranges of household costs (both direct and indirect) were not available, the range variability of Lambaréné was used. These two sites were used as they had the widest cost variation. In Lambaréné direct and indirect household savings ranges were estimated with bootstrapping techniques. Bootstrapping involves repeatedly taking random samples with replacement from a sample dataset in order to estimate the population statistics of the original sample [34], [35], [36]. Where ranges were unavailable we assumed the range to be of 25% less and more of the average value, specifically the proportion of children under 5 years of age with malaria accessing health facilities and rates of hospitalization with malaria parasites.

#### Threshold analysis

The point at which an intervention becomes cost effective remains debatable. The selection of cost-effectiveness thresholds in published literature is subjective [37]. Recent studies have used a multiple of per capita Gross National Income (GNI) and Gross Domestic Product (GDP) [38], [39], [40], [41], [42]. For this analysis we take the most conservative cut-off of USD 36 to reflect a *highly* cost-effectiveness intervention, and from USD 36 to USD 202 to reflect a cost effective intervention. As explained by Shillcutt and others [37] these thresholds are based on estimates by the World Bank in 1993 to recommend a minimum care package of services in low and middle income countries [43], and again in 1996 in an effort to define research priorities [44]. The committees specified USD150 per DALY as ‘attractive’ cost effectiveness and USD25 per DALY as ‘highly attractive’ cost effectiveness for low-income countries. For the purposes of our analysis these two thresholds where then inflated to their 2007 equivalent of USD 202 and USD 36 [45].

Multivariate threshold analysis was performed using the Goal Seek simulation (Palisade© @Risk add-in tool to Microsoft Excel©) to estimate at what level key input variables (IPTi efficacy, CFR, malaria incidence and cost per dose of IPTi delivered) cease to be *highly* cost effective (USD36). Threshold levels were stochastically estimated over 1000 simulations by varying all input parameters within the ranges assigned. The Pearson correlation coefficients, calculated on the simulations, present the magnitude of the relation between ICERs (in terms of DALYs averted) and the main parameters that determine them. The higher the coefficient (in absolute value) the more influence the variable has on the overall ICER. As the CFR is a major driver in DALY calculations and because precise site specific CFR were not available a further univariate sensitivity analysis was undertaken to assess their influence on the ICERs.

## Results

### Gross Intervention Costs

Table S4 presents the costs and cost savings associated with delivering IPTi. Gross intervention costs reflect the economic cost of delivering IPTi per 1000 infants, having taken into account site-specific drop out rates. The cost of the dose is largely determined by the cost of the drug. The cost of delivery of IPTi with SP in trial settings to 1000 infants ranged from USD 353 in Ifakara, Tanzania to USD 496 in Navrongo, Ghana. The alternative IPTi drug regimens cost more as the drugs cost more than SP, ranging from USD 1244 to deliver AQ3-AS3 in western Kenya to USD 4207 to deliver CD3 in Korogwe and Same, Tanzania.

### Net Intervention Costs

The net intervention costs (Table S4) reflect the savings to the formal health system due to less malaria inpatient and outpatient visits after accounting for the cost of delivering IPTi. Among the sites that had a statistically significant impact on reducing malaria, site specific analysis shows that there are health system cost savings in Ifakara (all the confidence intervals are highly negative which signifies that in all circumstances modeled the public health system benefits from cost savings), cost savings are likely in Navrongo and there is a reduction in health system costs in the other sites. When using the efficacy data from the individual trials and the IPTi-SP pooled analysis, all sites indicate health system cost savings or no increase of health system costs. Where IPTi did not have a statistically significant impact on malaria episodes (Lambaréné, Korogwe and Same using SP, western Kenya, Korogwe and Same using CD3, and Same using MQ), IPTi is likely to increase health system costs as malaria cases may not fall but there is the additional cost of the intervention. Same, Tanzania had a very low transmission, 10 fold less than predicted, therefore this trial arm was stopped early and was under-powered to detect a significant PE. The additional cost to the health system is most stark when the more expensive non-SP drugs are used and do not show a significant reduction in malaria.

### Cost Savings to Households

Household cost savings, both direct and indirect, show the potential societal economic impact of IPTi (Table S4). Results show that in study sites where IPTi has a statistically significant impact on malaria, considerable direct savings are made at the household level, ranging from USD 77 in Manhica with SP to USD 780 in western Kenya with AQ3-AS3 per 1000 infants. Indirect cost savings ranging from USD 91 in Tamale with SP to USD 1468 in western Kenya AQ3-AS3 per 1000 infants. Where IPTi was not shown to have a significant PE against malaria these savings will not be seen.

### The Cost Per Malaria Episode Averted

In sites where IPTi had a statistically significant effect on reducing malaria, the cost per episode averted for IPTi-SP is very low, USD 1.36–4.03 based on trial specific data and USD 0.68–2.27 based on the pooled analysis, see Table S4. For non SP IPTi, the lowest cost per case averted is USD 4.62 in western Kenya with AQ3-AS3 and the highest is Korowge with MQ at USD 18.56.

### The Costs Per DALY Averted

In sites where IPTi had a significant effect on reducing malaria, IPTi is *highly* cost-effective using SP (i.e. under USD 36 per DALY averted). In western Kenya, both AS3 IPTi drug combinations are highly cost effective, although AQ3-AS3 is more cost effective than SP+AS3. In Korogwe MQ is cost effective at USD 39.63 per DALY averted, see Table S4.

### Threshold and correlation analysis

 shows (for each site in which IPTi showed a statistically significant PE) at which levels of PE, CFR, incidence of malaria and intervention unit cost (this largely reflects the drug cost), that IPTi ceases to be a *highly* cost-effective intervention. In most of the cases, especially for the epidemiological/clinical factors (PE, incidence of malaria and CFR), the threshold of USD 36 per DALY averted is not reached within the ranges of each variable and according to the level of accuracy required for the calculation. The analysis shows, for instance, that in Kumasi, if the PE of SP dropped to 8%, the ICER would be USD 7.68 per DALY averted (actual threshold). In Manhica a drop of the CFR to 1% would lead to an ICER of USD 18.72, or if the incidence of malaria dropped to 0.09 the ICER would increase to USD 16.41.

The threshold simulation further shows that, apart from in Korogwe (with MQ), the cost of delivery of IPTi across the settings could increase considerably and still be *highly* cost effective. For instance, in the case of Navrongo, there would need to be an increase to USD 1.52 per dose delivered using site specific data and USD 1.92 per dose using data from the IPTi-SP pooled analysis, before IPTi stopped being *highly* cost effective; the actual cost is USD 0.13 per dose. IPTi using alternative antimalarials to SP is closer the threshold of USD 36. Alternative drugs are much more expensive than SP.

Summarizing the results from Table S5 shows that the clinical and epidemiological variables (PE, incidence of malaria and CFR), affect CE ratios much more than intervention costs.

Table S6 shows (i) the level of the CFR at which the intervention is no longer highly cost-effective, even if outside 1–3% range used in the PSA and (ii) the value of the ICERs if the CFR is set at the extremely low level of 0.1%. In most cases the intervention would be highly cost-effective even with a CFR as low at 0.35%. The IPTi SP trials in Ifakara, Kumasi, Navrongo and Tamale, would be borderline highly cost-effective with a CFR as low as 0.1%.

## Discussion

In studies where IPTi was shown to have a statistically significant impact on reducing malaria, it was cost effective in all sites with all drugs and highly cost effective in all but one site that used MQ. As mentioned previously, the thresholds that have been used in the literature to determine highly cost effective interventions and cost-effectiveness interventions vary. In this analysis using the most conservative cut-off points, IPTi is highly cost effective in the majority of studies. Had we chosen the WHO threshold of under 1× Gross Domestic Product per capita, all of the studies that had a statistically significant impact on malaria would have been considered well within the highly cost effective range [46].

Although not part of this analysis, if we were to add the benefits of the additional health gains and subsequent cost savings from averting anaemia and those associated with averting the non-malaria admissions included in ‘all-cause’ hospitalisations (here we included only hospital admissions with parasitaemia), the ICERS would be even more cost effective. For example, IPTi with SP was not seen to have a statistically significant impact on clinical malaria in the trial in Gabon. This was due to a number of reasons, including a steady decline in the malaria incidence in Lambaréné area over the past decade (unpublished data), the high mobility of the local population and a study design with a close-knit passive and active follow-up system that led to the creation of an outstandingly healthy study cohort [15]. However, Lambaréné did show a 26% (0%, 45%) PE against moderate anaemia in the first year of life, the benefits of which are not measured in this analysis.

Cost effectiveness analysis aims to inform policy makers on the cost-effectiveness associated with different interventions when decisions have to be made about where to allocate limited funds. However, caution should be exercised when comparing the cost-effectiveness of different malaria control strategies [12] as there needs to be an understanding of site specific epidemiological and health system characteristics, the costing perspective and how different malaria control strategies complement and/or substitute one another. With this in mind, delivery cost of IPTi was between USD 0.13 (per dose of SP in southern Tanzanian and Ghana) to USD 1.92 (per dose of CD at 3 days each dose in northern Tanzania). Other malaria prevention strategies have reported annual costs (also adjusted to USD 2007) of providing insecticide treated nets (ITNs) of USD 1.40–USD 3.85 [47], USD 3.42–USD 5.83 for indoor residual household spraying [48], USD 1.94 to deliver IPT to school children (3 doses, SP & AQ3) [49], and USD 2.60 when delivering a full course of IPT to pregnant women (2 doses, IPTp-SP) via community care and USD2.30 via health centres [50].

The incremental benefit of IPTi in addition to ITN use needs to be explored further [51]. In the sites included in this analysis ITN ownership and use varied. For example in western Kenya, ITNs were provided alongside the timing of IPTi, thus the PE of IPTi was in the context of high ITN use [6], whereas in Manhica ITN use was zero at the time of the study [14].

The potential impact of IPTi on the EPI needs careful consideration: will it overburden EPI activities and lead to inequities [52], [53] or conversely will the additional benefits of IPTi provide extra resources and momentum that will strengthen the EPI and increase vaccination uptake? The level of EPI coverage will also impact the ICERs as there are certain fixed costs that remain constant regardless of EPI coverage and the subsequent number of IPTi doses given (such as communication and sensitisation materials and a minimum number of training workshops) and certain variable costs that are related to coverage (such as IPTi drugs dispensed).

For the multi-dose IPTi drug regimens used in western Kenya, Korogwe and Same, additional costs were incurred delivering day two and three doses to achieve maximal efficacy. In a bid to reflect effectiveness rather than trial efficacy the costs of research staff used as adherence monitors were excluded from this analysis. It is important to recognise that there is likely to be a gap between trial efficacy and programmatic effectiveness for multi-day regimens. The threshold analysis shows the scope for additional IPTi delivery costs associated with monitoring adherence or a potential fall in protective efficacy if day two and three IPTi doses are not taken. For example, in western Kenya the threshold analysis presented in Table S5 shows that the PE of IPTi with SP-AS3 could fall from the trial level of 22% to 9% and from 25% to 10% with AQ3-AS3 and still remain highly cost effective. Alternatively, the IPTi cost per dose would need to increase from USD 0.60 to 1.33 with SP-AS3 and USD 0.44 to 1.64 with AQ3-AS3 before it was no longer highly cost effective. The costs and effects of using community health workers to prompt caretakers to administer IPTi doses in days 2 and 3 of multi dose IPTi regimens still need to be evaluated.

Every effort was made to conduct a rigorous analysis, but some limitations remain. Costing the intervention was a challenge as we had to extrapolate data from Mtwara, Tanzania to other settings and countries. The use of PPP adjustments is a recognised approach [29], but it would have been advantageous to look at cost variation across sites using primary data. However, the other sites in our analysis were randomized control trials and therefore it would not have been possible to measure real system delivery costs. Cost variation, within and across countries, has important implications for planning health services and budgets, however there is surprisingly little data published on this topic [54]. A within-country cost and cost-effectiveness analysis of nationwide school-based helminth control in Uganda showed substantial variation between six districts in the cost per individual treated (USD0.41–USD0.91)[55]. Hutton and others investigated variation of maternity costs in Thailand and Cuba in the context of multicountry, multicentre randomised controlled trials. Unit costs per antenatal visit and per pregnancy showed considerable variation, due largely to staffing patterns and productivity [56]. Across 5 Sub-Saharan African countries, the annualized economic costs per ITN distributed varied from USD2.75 in Togo to USD8.05 in Senegal [57], explained mainly by differences in the composition of each programme, levels of existing resources and spare capacity.

The implications of cost variation for decision making depend critically on the cost-effectiveness thresholds applied [58]. By undertaking a threshold analysis, using a particularly conservative threshold of US$36, we were able to show that the cost per dose of IPTi, especially IPTi-SP, could vary, more specifically increase considerably, and still remain highly cost effective across most of the settings.

To be consistent with Hutton et al (2009) DALYs were calculated with no age weighting, however we recognize that the debate on the use of age weighting continues [59], [60]. Supporters of age weighting suggest all societies have age-based biases when deciding resource allocation. Detractors suggest DALYs can be criticized on equity grounds as every year of life is of equal value a priori, and on empirical grounds as the standard age weights may not accurately reflect social values.

All the ICERs presented in this analysis reflect the PE during the intervention period and using the pooled IPTi-SP PE. The analysis does not present the potential cost implications of an increase in drug resistance which is likely to lead to other health, health system and household costs [61], [62]. The threshold analysis presented here shows that there is room for the PE to fall and still remain highly cost effective in all sites where IPTi had a statistically significant effect on clinical malaria.

While IPTi is shown to be low cost and highly cost effective in this analysis, this does not guarantee that it will be adopted as a strategy. The funding of the intervention is vital and given the scarce resources and competing interventions (malaria and non-malaria related) countries may recognise the advantages of introducing IPTi but struggle to secure the funds. One of the great advantages of delivering IPTi is that it relies on an existing, well established delivery strategy such as the EPI scheme that already reaches a high proportion of the target IPTi recipients across all malarious countries.

Given the limited public health expenditure in many low income countries, decision makers need cost-effectiveness data to prioritise potential interventions for scale-up. This analysis shows that in many settings IPTi is a highly cost effective intervention, and that IPTi-SP would remain highly cost effective even if the level of PE of the intervention or the malaria incidence or the CFR were to decline. IPTi benefits from an already existing delivery system, EPI, which is a routine point of contact for many infants, making IPTi potentially one of the most cost effective malaria interventions available in areas where malaria transmission is moderate to high.

## Supporting Information

Table S1 Characteristics of intermittent preventive treatment in infants (IPTi) trials with economic data.
(0.07 MB DOC)

Table S2 Cost-Effectiveness Analysis model: Health Seeking and Effectiveness Inputs.
(0.06 MB DOC)

Table S3 Cost-Effectiveness Analysis Model: Cost Inputs (USD 2007).
(0.05 MB DOC)

Table S4 Cost-Effectiveness Ratios and savings with range (95%) from Monte-Carlo Simulation (1000 iterations).
(0.07 MB DOC)

Table S5 Correlation of Cost-Effectiveness Ratios and threshold levels of several variables (Monte Carlo simulations, 1000 iterations).
(0.05 MB DOC)

Table S6 One way sensitivity analysis Case Fertility Rate.
(0.04 MB DOC)
