# Supplementary material for: The Cost-Effectiveness of Intermittent Preventive Treatment for Malaria in Infants in Sub-Saharan Africa
Source: PLoS One. 2010 Jun 15;5(6):e10313. doi: 10.1371/journal.pone.0010313 (PMC2886103; doi:10.1371/journal.pone.0010313)
Supplement: Table S1 — Characteristics of intermittent preventive treatment in infants (IPTi) trials with economic data. (0.07 MB DOC) [file pone.0010313.s001.doc]

**Table 1: Characteristics of intermittent preventive treatment in infants (IPTi) trials with economic data**

| ***Study*** ***Site*** | ***Study Period*** | ***Transmission & (Entomological Inoculation Rate /Year)*** | ***Use of Insecticide Treated Nets (%)*** | ***No of infants in placebo/received at least one dose of IPTi Drug*** | ***Age at drug dosing*** | ***Site specific PE -Intervention time and primary outcome - used in CEA model*** |
| --- | --- | --- | --- | --- | --- | --- |
| Ifakara,Tanzania[13] | 8/1999 – 4/2001 | Perennial (29) | 67 | Placebo 351 | 2, 3, 9 months (at time of DPT2 ^c^, DPT3 & measles) | All episodes of clinical malaria up to 12 months of age |
|  |  |  |  | SP = 350 |  |  |
| Navrongo, Ghana [18] | 9/2000–6/2004 | Highly seasonal (418) | 18 | Placebo =1,225 | 3, 4, 9, 12 (at time of DPT2, DPT3, measles + extra at 12 months) | All episodes of clinical malaria up to 15 months of age |
| Manhiça, Mozambique [14] | 9/2002 – 2/2004 | Perennial with seasonal peaks (38) | 0 | Placebo= 755 | 3, 4, 9 Months (at time of DPT2, DPT3 & measles) | All episodes of clinical malaria up to 12 months of age |
|  |  |  |  | SP = 748 |  |  |
| Kumasi, Ghana[17] | 1/2003–9/2005 | Perennial with seasonal peaks (400) | 1.6 | Placebo= 535 | 3, 9, 15 (at time of DPT3 & measles + extra at 15 months) | All episodes of clinical malaria (>500 parasites/ul) up to 18 months of age |
|  |  |  |  | SP =535 |  |  |
| Tamale, Ghana [16] | 3/2003–7/2005 | Perennial with seasonal peaks (NA) | 3 | Placebo= 600 | 3, 9, 15 (at time of DPT3 & measles + extra at 15 months) | All episodes of clinical malaria up to 18 months of age |
|  |  |  |  | SP =600 |  |  |
| Lambaréné, Gabon [15] | 12/2002 –8/2006 | Perennial with seasonal peaks (50) | 5 | Placebo=504 | 3, 9, 15 months (at time of DPT3 & measles + extra visit at 15 months) | All episodes of clinical malaria up to 18 months of age |
|  |  |  |  | SP = 507 |  |  |
| Western Kenya [6] | 3/2004 – 3/2007 | Perennial (7) | 57 ^a^ | Placebo = 337 | 2, 3, 9 months (at time of DPT2, DPT3 & measles) | All episodes of clinical malaria up to 12 months of age |
|  |  |  |  | SP-AS3 = 339 |  |  |
|  |  |  |  | AQ3-AS3 = 347 |  |  |
|  |  |  |  | CD3 = 342 |  |  |
| Korogwe (K) & Same (S), Tanzania [5] | 2004-2008 | K: Perennial with seasonal peak | K: 88 ^b^ | K / S | 2, 3, 9 months (at time of DPT2, DPT3 & measles) | All episodes of clinical malaria up to 11 months of age |
|  |  |  |  | Placebo= 320 / 284 |  |  |
|  |  | S: Low (NA) | S: 88 | SP = 319 / 283 |  |  |
|  |  |  |  | CD3= 317 / 285 |  |  |
|  |  |  |  | MQ = 320 / 284 |  |  |

Table adapted from [4], Grobusch M, Egan A, Gosling RD, Newman R (2007) Intermittent preventive therapy for malaria: progress and future directions. Current Opinion in Infectious Diseases 20: 613-620 and site specific publications, (a) 2003 Kenyan Demographic Health Survey (b) JF Mosha (Unpublished) (c) diphtheria-tetanus toxoid–pertussis vaccine
