# Supplementary material for: The Cost-Effectiveness of Intermittent Preventive Treatment for Malaria in Infants in Sub-Saharan Africa
Source: PLoS One. 2010 Jun 15;5(6):e10313. doi: 10.1371/journal.pone.0010313 (PMC2886103; doi:10.1371/journal.pone.0010313)
Supplement: Table S2 — Cost-Effectiveness Analysis model: Health Seeking and Effectiveness Inputs. (0.06 MB DOC) [file pone.0010313.s002.doc]

**Table 2: Cost-Effectiveness Analysis Model: Health Seeking and Effectiveness Inputs**

| ***CEA Model Assumptions Best Estimates and (Ranges used in the sensitivity analysis)*** | | | | | | | | |
| --- | --- | --- | --- | --- | --- | --- | --- | --- |
| ***Study Site*** | ***IPTi Drug*** | ***Using Site Specific Estimates*** ^a^ | | ***Using SP Pooled Analysis*** ^b^ | | ***Malaria episodes accessing government facilities (%)*** | ***Case Fatality Rate (%)*** | ***Cases hospitalised with malaria parasites – proxy for proportion of malaria cases that develop into severe malaria*** |
|  |  | ***IPTi Protective Efficacy***  ***(%)*** ***Reduction in All Episodes of Clinical Malaria During Intervention Period*** | ***Malaria incidence***  ***(taken from placebo group for same duration as IPTi Protective Efficacy)*** | ***Combined estimate using random effects meta-analysis*** ***up to 12 months of age*** | ***Incidence of malaria up to 12 months of age*** |  |  |  |
| Ifakara,Tanzania | SP | 62.3 (44.2, 74.6) | 0.43 (0.27,0.64) |  | 0.54 (0.40,0.67) | 55 ^c^ (41, 69) |  | 0.17 ^b^ (0.13, 0.21) |
| Navrongo, Ghana | SP | 24.9 (14.5, 34.1) | 1.10 (0.55,1.62) |  | 1.10 (0.82,1.37) | 28 ^d^ (21, 35) |  | 0.13 ^b^ (0.10, 0.16) |
| Manhiça, Mozambique | SP | 22.6 (1.6, 39.2) | 0.55 (0.28,0.82) | 30.3% (19.8%; 39.4%) | 0.79 (0.59,0.99) | 51 ^e^ (38, 64) |  | 0.13 ^b^ (0.10, 0.16) |
| Kumasi,Ghana | SP | 20.3 (10.6, 28.9) | 1.20 (0.65,1.93) |  | 1.27 (0.96,1.59) | 28 ^d^ (21, 35) |  | 0.05 ^b^ (0.04, 0.06) |
| Tamale, Ghana | SP | 22.5 (11.8, 31.9) | 1.16 (0.58,1.74) |  | 0.95 (0.71,1.19) | 28 ^d^ (21, 35) |  | 0.02 ^b^ (0.05, 0.07) |
| Lambaréné,Gabon | SP | 17 (-24, 44) | 0.16 (0.08,0.24) |  | 0.16 (0.08,0.24) | 40 ^f^ (30,50) |  | 0.13  ^b^ (0.10, 0.16) |
| Western Kenya | SP + AS3 | 22.2 (2.5,37.8) |  |  |  |  | 1.57 ^h^ (1.00, 3.00) |  |
|  | AQ3 + AS3 | 24.7 (6.4,39.5) | 1.33 (0.67,1.99) |  |  | 37 ^g^ (10, 50) |  | 0.22 ^b^ (0.17, 0.28) |
|  | CD3 | 10.5 (-11.6,28.2) |  |  |  |  |  |  |
| Korogwe, Tanzania | SP | -6.7 (-45.9, 22.0) |  | NA | NA |  |  |  |
|  | CD3 | 10.8 (-24.6, 36.1) | 0.31 (0.16,0.46) |  |  |  |  | 0.15 ^a^ (0.105,0.175) |
|  | MQ | 38.1 (11.8, 56.5) |  |  |  | 55 ^c^ (41, 69) |  |  |
| Same, Tanzania | SP | -77.2 (-505.4, 48.1) |  |  |  |  |  |  |
|  | CD3 | -52.5 (-440.6, 57.0) | 0.02 (0.01,0.03) |  |  |  |  | 0.25^a^ (0.1, 0.4) |
|  | MQ | 50.2 (-171.9, 90.9) |  |  |  |  |  |  |

NA Not applicable, (a) Site specific publication, (b), Figure given in [32], (c) Matovu personal communication, (d) DHS Ghana 2003, (e) DHS Mozambique 2003, (f) DHS Gabon 2003, (g) CDC Kisumu Household Survey Data, (h) [24]
