# Supplementary material for: The Cost-Effectiveness of Intermittent Preventive Treatment for Malaria in Infants in Sub-Saharan Africa
Source: PLoS One. 2010 Jun 15;5(6):e10313. doi: 10.1371/journal.pone.0010313 (PMC2886103; doi:10.1371/journal.pone.0010313)
Supplement: Table S3 — Cost-Effectiveness Analysis Model: Cost Inputs (USD 2007). (0.05 MB DOC) [file pone.0010313.s003.doc]

**Table 3: Cost-Effectiveness Analysis Model: Cost Inputs (USD 2007)**

| ***Study Site*** | ***IPTi Cost Per Dose Delivered*** | ***Provider Malaria***  ***Treatment Costs*** | | ***Household Malaria***  ***Treatment Cost***  ***(Direct)*** | | ***Household Malaria***  ***Treatment Cost***  ***(Indirect)*** | |
| --- | --- | --- | --- | --- | --- | --- | --- |
|  |  | Uncomplicated/ Outpatient | Severe/ Inpatient | Uncomplicated/ Outpatient | Severe/ Inpatient | Uncomplicated/ Outpatient | Severe/ Inpatient |
| Ifakara,Tanzania | **SP** = 0.13 (0.09,0.17) | 4.33 (3.79,4.84) | 17.60 (13.44,21.76) | 1.18 (0.68,1.74) | 5.32 (4.73,5.91) | 3.04 (1.87,4.20) | 13.94 (5.77,22.11) |
| Navrongo,Kumasi, Tamale, Ghana | **SP** = 0.13 (0.09,0.17) | 2.64 (1.86,3.42) | 25.21 (19.26,31.16) | 4.18 (3.73,4.63) | 28.72 (25.54,31.90) | 0.92 (0.53,1.30) | 17.57 (7.27,27.87) |
| Manhiça, Mozambique | **SP** = 0.13 (0.09,0.17) | 3.78 (2.66,4.90) | 8.96 (6.84,11.07) | 0.84 (0.42,1.41) | 3.49 (2.62,4.45) | 1.57 (1.31,1.88) | 4.89 (4.31,5.56) |
| Lambaréné,Gabon | **SP** = 0.17 (0.12,0.22) | 14.42 (9.56,18.93) | 48.50 (37.50,59.95) | 13.10 (5.28,22.45) | 38.19 (32.42,43.96) | 4.61 (2.56,7.05) | 10.10 (4.49,15.71) |
| Western Kenya | **SP + AS3 =** 0.60 (0.42,0.78) |  |  |  |  |  |  |
|  | **AQ3 + AS3 =** 0.44 (0.31,0.57) | 2.82 (1.66,3.88) | 21.81 (14.59,29.76) | 1.17 (0.49,1.98) | 8.46 (4.46,12.28) | 7.72 (3.74,11.17) | 30.22 (11.77,49.57) |
|  | **CD3 =** 1.49 (1.04,1.94) |  |  |  |  |  |  |
| Korogwe & Same, Tanzania | **SP** = 0.13 (0.09,0.17) |  |  |  |  |  |  |
|  | **CD3** = 1.48 (1.04,1.92) | 4.33 (3.79,4.84) | 17.60 (13.44,21.76) | 1.18 (0.68,1.74) | 5.32 (4.73,5.91) | 3.04 (1.87,4.20) | 13.94 (5.77,22.11) |
|  | **MQ** = 0.63 (0.44,0.82) |  |  |  |  |  |  |
