# Supplementary material for: The Cost-Effectiveness of Intermittent Preventive Treatment for Malaria in Infants in Sub-Saharan Africa
Source: PLoS One. 2010 Jun 15;5(6):e10313. doi: 10.1371/journal.pone.0010313 (PMC2886103; doi:10.1371/journal.pone.0010313)
Supplement: Table S4 — Cost-Effectiveness Ratios and savings with range (95%) from Monte-Carlo Simulation (1000 iterations). (0.07 MB DOC) [file pone.0010313.s004.doc]

**Table 4: Cost-Effectiveness Ratios and savings with range (95%) from Monte-Carlo Simulation (1000 iterations)**

|  | ***Total Episodes Averted*** | | ***Cost-Effectiveness Ratios*** | | ***IPTi Costs per 1000 hypothetical infants ^b^*** | ***Savings from fewer malaria cases for 1000 hypothetical infants entering the model*** | | |
| --- | --- | --- | --- | --- | --- | --- | --- | --- |
|  | ***Malaria Episodes Averted*** | ***DALYs averted*** | ***Cost per malaria episode averted*** | ***Cost per DALY averted*** | ***Gross Intervention Costs ^c^*** | ***Net Intervention Costs***  ***(IPTi implementation costs – health system savings)*** | ***Total Household direct treatment cost savings*** | ***Total Household indirect treatment cost savings*** |
| ***Studies using SP*** | | | | | | | | |
| Ifakara, Tanzania* |  |  |  |  |  |  |  |  |
| Trial | 270 (188,364) | 133 (77,207) | 1.36 (0.90,1.96) | 2.90(1.61,4.70) | 353 (281,426) | -735 (-1189,-348) | 310 (195,457) | 804 (490,1193) |
| Pooled | 160 (118,207) | 77(47,119) | 2.27 (1.56,3.17) | 4.85 (2.79,7.72) |  | -248 (-471, -50) | 170 (112,241) | 442 (285,631) |
| Navrongo, Ghana * |  |  |  |  |  |  |  |  |
| Trial | 270 (162,394) | 139 (73,229) | 1.50 (0.90,2.39) | 3.05 (1.56,5.32) | 496 352, 653) | -72 (-320,138) | 598 (344,906) | 242 (124,395) |
| Pooled | 327 (179, 518) | 169 (62, 380) | 1.18 (0.58, 2.48) | 2.41 (0.76, 6.85) | 376 (266, 494) | -167 (-717, 244) | 727 (270, 1392) | 294 (95, 686) |
| Manhiça, Moz * |  |  |  |  |  |  |  |  |
| Trial | 116 (40,203) | 57 (18,110) | 4.03 (1.71,9.18) | 8.63 (3.2219.98) | 363 (286,435) | 69 (-179,281) | 77 (24,144) | 131 (44,234) |
| Pooled | 236 (173, 304) | 116 (70,175) | 1.58 (1.08,2.21) | 3.39 (1.95,5.39) |  | -189 (-423,8) | 139 (85,206) | 242 (167,330) |
| Kumasi, Ghana* |  |  |  |  |  |  |  |  |
| Trial | 239 (138,358) | 123 (61,206) | 1.66 (0.96,2.73) | 3.39 (1.68,6.15) | 366 (291,444) | 104 (-63,250) | 376 (208,579) | 120 (62,195) |
| Pooled | 379 (206, 599) | 195 (75,444) | 0.68 (0.34,1.40) | 1.39 (0.46, 3.43) | 251 (178, 330) | -156 (-579, 115) | 587 (271, 1132) | 185 (64,457) |
| Tamale, Ghana * |  |  |  |  |  |  |  |  |
| Trial | 256 (149,380) | 132 (67,221) | 1.55 (0.91,2.42) | 3.16 (1.59,5.58) | 366 (291,445) | 141 (-8,273) | 341 (190,529) | 91 (47,148) |
| Pooled | 283 (157, 451) | 146 (55, 346) | 0.91 (0.45,1.91) | 1.85 (0.68,5.31) | 249 (177, 328) | 9 (-287, 177) | 387 (182, 690) | 107 (44,209) |
| Lambaréné, Gabon |  |  |  |  |  |  |  |  |
| Trial | 20 (-20,57) | 10 (-10,32) | 11.93 (-90.60,102.87) | 29.94 (-184.16, 208.75) | 426 (339,509) | 263 (-77,561) | - | - |
| Pooled | 66 (25, 120) | 34 (8, 87) | 5.05 (2.05,3.95) | 10.1 (2.99,44.86) | 311 (219, 398) | -188 (-817, 179) | 439 (139,1127) | 146 (48,366) |
| Korogwe, Tanzania ^a^ |  |  |  |  |  |  |  |  |
| Trial | -32 (-111,37) | -16 (-56,18) | -15.72 (-60.17,53.18) | -37.21 (-131.37,111.18) | 372 (296,449) | 490 (223,796) | - | - |
| Pooled | 92 (58,131) | 45 (25,73) | 4.27 (2.67,6.57) | 9.11 (4.83,15.31) |  | 25 (-137,171) | - | - |
| Same, Tanzania ^a^ |  |  |  |  |  |  |  |  |
| Trial | -36 (-84,-2) | -18 (-43,-1) | -0.71,(-72.10, 3.59) | -7.34 (-154.88,-6.83) | 375 (298,453) | 545 (362,794) | - | - |
| Pooled | 6 (4,9) | 3 (2,5) | 67.00 (41.17,105.13) | 143.78 (76.14,246,41) |  | -952 (-1661,-346) | - | - |
| ***Non SP Study Drugs*** | | | | | | | | |
| Western Kenya |  |  |  |  |  |  |  |  |
| *Trial SP-ASt3 | 277 (104,479) | 140 (48,261) | 7.72 (3.44,16.66) | 16.09 (6.27,36.44) | 1714 (1361.,2062) | 1025 (288, 1633) | 689 (213, 1347) | 1296 (396,2587) |
| *Trial AQ3-AS3 | 313 (143,513) | 158 (65,285) | 4.62 (2.31,8.87) | 9.63 (4.21,19.23) | 1244 (994,1498) | 464 (-263, 1029) | 780 (282, 1462) | 1468 (506,2799) |
| Trial CD3 | 120 (-65,311) | 61 (-31, 166) | 11.01 (-152.50, 78.40) | 39.22 (-325.20, 385.57) | 4113 (3264,4962) | 3814 (2850, 4782) | - | - |
| Korogwe,Tanzania |  |  |  |  |  |  |  |  |
| *Trial MQ | 110 (54,174) | 54 (24,95) | 18.56 (9.81,33.67) | 39.63 (18.09,76.58) | 1802 (1430,2170) | 1390 (946,1819) | 117 (54,196) | 303 (141,510) |
| Trial CD3 | 23 (-44,88) | 11 (-21,45) | 130.01 (-600.97,669.14) | 243.80 (-1317.69,419.24) | 4207 (3346,5073) | 4121 (3220,5017) | - | - |
| Same,Tanzania |  |  |  |  |  |  |  |  |
| Trial MQ | -2 (-24,14) | -1 (-12,7) | -211.29 (-1054.29,1163.82) | -275.30 (-2317.29,2455.81) | 1819 (1444,2190) | 1828 (1446,2212) | - | - |
| Trial CD3 | -29 (-71,1) | -14 (-38,0.5) | -308.63 (-1092.65, 637.79) | -633.82 (-2299.85, 1254.10) | 4207 (3353,5066) | 4347 (3480,5225) | - | - |

* Statistically significant effect on malaria

^a^ Same and Korogwe were not part of the Pooled Analysis. All input parameters have been inserted as probability distributions (epidemiological variables as well as costs)

^b^ Taking into account trial drop out rates

***^c^*** Trial Gross Intervention Costs include delivering all IPTi doses, where different, pooled Gross Intervention Costs reflect the cost of delivering IPTi doses up to 12 months
