# Supplementary material for: The Cost-Effectiveness of Intermittent Preventive Treatment for Malaria in Infants in Sub-Saharan Africa
Source: PLoS One. 2010 Jun 15;5(6):e10313. doi: 10.1371/journal.pone.0010313 (PMC2886103; doi:10.1371/journal.pone.0010313)
Supplement: Table S5 — Correlation of Cost-Effectiveness Ratios and threshold levels of several variables (Monte Carlo simulations, 1000 iterations). (0.05 MB DOC) [file pone.0010313.s005.doc]

**Table 5: Correlation of Cost-Effectiveness Ratios and threshold levels of several variables (Monte Carlo simulations, 1000 iterations)**

|  |  | **PE** | | | **CFR** | | | **Incidence of malaria** | | | **IPTi intervention costs (per dose)** | | |
| --- | --- | --- | --- | --- | --- | --- | --- | --- | --- | --- | --- | --- | --- |
| Study Site |  | Pearson Correlation Coefficient* | Variable  Threshold | Actual Threshold USD | Pearson Correlation Coefficient* | Threshold - Simulation | Actual Threshold USD | Pearson Correlation Coefficient* | Threshold - Simulation | Actual Threshold USD | Pearson Correlation Coefficient* | Threshold - Simulation | Actual Threshold USD |
| Ifakara SP | Trial | -0.33 | 0.24 | 7.18 | -0.64 | 0.01 | 6.73 | -0.52 | 0.19 | 7.05 | 0.36 | 1.51 | 36.00 |
| Ifakara SP | Pooled | -0.44 | 0.13 | 11.23 | -0.69 | 0.01 | 10.58 | -0.33 | 0.23 | 11.31 | 0.39 | 0.96 | 36.00 |
| Navrongo SP | Trial | -0.45 | 0.10 | 7.00 | -0.55 | 0.01 | 6.67 | -0.55 | 0.47 | 6.86 | 0.32 | 1.52 | 36.00 |
| Navrongo SP | Pooled | -0.45 | 0.13 | 5.58 | -0.69 | 0.01 | 5.27 | -0.31 | 0.46 | 5.63 | 0.40 | 1.92 | 36.00 |
| Manhica SP | Trial | -0.66 | 0.09 | 16.43 | -0.26 | 0.01 | 18.72 | -0.28 | 0.09 | 16.41 | 0.16 | 0.55 | 36.00 |
| Manhica SP | Pooled | -0.45 | 0.13 | 7.83 | -0.68 | 0.01 | 7.39 | -0.31 | 0.33 | 7.90 | 0.40 | 1.40 | 36.00 |
| Kumasi SP | Trial | -0.51 | 0.08 | 7.68 | -0.55 | 0.01 | 7.40 | -0.57 | 0.51 | 7.64 | 0.30 | 1.38 | 36.00 |
| Kumasi SP | Pooled | -0.43 | 0.13 | 3.21 | -0.68 | 0.01 | 3.04 | -0.32 | 0.54 | 3.24 | 0.45 | 3.34 | 36.00 |
| Tamale SP | Trial | -0.49 | 0.09 | 7.18 | -0.52 | 0.01 | 6.91 | -0.54 | 0.49 | 7.13 | 0.31 | 1.47 | 36.00 |
| Tamale SP | Pooled | -0.43 | 0.13 | 4.27 | -0.68 | 0.01 | 4.04 | -0.36 | 0.40 | 4.31 | 0.40 | 2.52 | 36.00 |
| Lambaréné SP | Pooled | -0.36 | 0.13 | 23.60 | -0.61 | 0.01 | 22.27 | -0.58 | 0.09 | 22.95 | 0.33 | 0.60 | 36.00 |
| Western Kenya SP+Art | Trial | -0.68 | 0.09 | 31.78 | -0.31 | 0.01 | 35.44 | -0.31 | 0.57 | 36.00 | 0.17 | 1.33 | 36.00 |
| Western Kenya AQ+Art | Trial | -0.65 | 0.10 | 20.42 | -0.41 | 0.01 | 21.03 | -0.40 | 0.56 | 21.79 | 0.22 | 1.64 | 36.00 |
| Korogwe MQ | Trial | -0.63 | 0.36 | 36.00 | -0.45 | 0.02 | 36.00 | -0.43 | 0.33 | 36.00 | 0.25 | 0.57 | 36.00 |
