# Supplementary material for: The Cost-Effectiveness of Intermittent Preventive Treatment for Malaria in Infants in Sub-Saharan Africa
Source: PLoS One. 2010 Jun 15;5(6):e10313. doi: 10.1371/journal.pone.0010313 (PMC2886103; doi:10.1371/journal.pone.0010313)
Supplement: Table S6 — One way sensitivity analysis Case Fertility Rate. (0.04 MB DOC) [file pone.0010313.s006.doc]

**Table 6: One way sensitivity analysis Case Fertility Rate**

| **Study Site** |  | **Cut–off level of CFR for ICERs to reach USD36** | **ICER if CFR = 0.1%** |
| --- | --- | --- | --- |
| Ifakara SP | Trial | 0.10% | 36.40 |
| Ifakara SP | Pooled | 0.19% | 59.60 |
| Navrongo SP | Trial | 0.10% | 36.02 |
| Navrongo SP | Pooled | 0.08% | 29.60 |
| Manhica SP | Trial | 0.28% | 82.81 |
| Manhica SP | Pooled | 0.13% | 43.00 |
| Kumasi SP | Trial | 0.11% | 39.36 |
| Kumasi SP | Pooled | 0.06% | 24.92 |
| Tamale SP | Trial | 0.10% | 36.78 |
| Tamale SP | Pooled | 0.05% | 22.73 |
| Lambaréné SP | Pooled | 0.65% | 173.95 |
| Western Kenya SP+Art | Trial | 0.28% | 82.31 |
| Western Kenya AQ+Art | Trial | 0.35% | 102.87 |
| Korogwe MQ | Trial | 1.57% | 425.40 |
